# Sleep Quality and Physical Activity of Night Shift Nurses Working at Hospitals: A Cross-Sectional Survey Study

**DOI:** 10.3390/nursrep16070223

**Published:** 2026-06-27

**Authors:** Vilma Zydziunaite

**Affiliations:** Department of Nursing and Social Welfate, Faculty of Health Sciences, Klaipedos Valstybine Kolegija/Higher Education Institution, 91274 Klaipeda, Lithuania; v.zydziunaite@kvk.lt

**Keywords:** hospital, International Physical Activity Questionnaire, night shift nurse, nursing, physical activity, Pittsburgh Sleep Quality Index, sleep quality, statistics, survey

## Abstract

**Background/Objectives**: The aim of the study was to explore the specific relationships between sleep quality, physical activity levels, and demographic characteristics (age, education level, and work experience) in night shift nurses working at hospitals. Understanding these relationships is critical for developing evidence-based scheduling, educational initiatives for sleep hygiene, and physical activity programs that can mitigate the negative impacts of night work, ultimately promoting nursing workforce sustainability and safer patient health outcomes. **Methods**: A cross-sectional design was implemented, involving 400 night shift nurses. Data were collected using a questionnaire, which included an Individual Characteristics Form, the Pittsburgh Sleep Quality Index and the International Physical Activity Questionnaire—Short Form. **Results**: The sleep quality of night shift nurses differed statistically significantly by age in almost all sub-scales (*p* < 0.001) except for the sub-scale “habitual sleep efficiency”. A significant difference was found across groups, with sleep latency (*p* = 0.038 *) increasing as work experience grew. Nurses with more experience utilized sleep medications (*p* = 0.014 *) more frequently than less experienced ones. Physical activity levels differed significantly (*p* < 0.05): the youngest group showed a higher proportion of high physical activity (52.3%) compared to the oldest (28.9%). A statistically significant negative correlation was found between total physical activity and the total PSQI score (r = −0.162, *p* = 0.001). **Conclusions**: The data show that night shifts consistently disrupt nurses’ sleep, and advancing age further compounds these difficulties. The elevated total Pittsburgh Sleep Quality Index (PSQI) scores verify severe sleep disruption across the sample. Physical activity operates as a beneficial behavioral modifier that significantly relates to better sleep quality, lower sleep latency, and less frequent use of sleep medications among night shift nurses.

## 1. Introduction

Night shift work is a key aspect of nursing within healthcare, ensuring continuous patient care [1]. Sleep is a fundamental necessity for human health, and sleep disorders, which are widespread worldwide, not only lead to a poorer quality of life but also contribute to mental and physical health problems of night shift nurses [2]. However, one of the most common problems faced by night shift nurses is their tendency to experience circadian rhythm disequilibrium [3]. Globally, night shift nurses often work rotating or permanent night shifts, leading to circadian desynchrony, and report chronic lack of sleep, severe fatigue, insomnia, and daytime dysfunction [4,5,6]. Poor sleep quality has significant professional and personal ramifications for nurses as it increases the risk of work-related errors, directly compromising patient safety and quality of care [4,6]. Night shift work is associated with increased risks of cardiovascular disease, metabolic syndrome, and mental health issues such as anxiety, depression, and burnout among night shift nurses [5]. While physical activity is widely recognized as a booster for sleep quality and physical health, the demanding nature of nursing—especially on night shifts—leads to sedentary lifestyles and poor diet, creating a cycle of fatigue and reduced well-being [7].

Physical activity includes a variety of body movements performed by skeletal muscles that require energy. It can be any form of movement, including leisure activities or work-related activities. Both moderate- and vigorous-intensity physical activity contribute to improved health [8]. Despite studies showing that nurses walk approximately eight kilometers during a ten-hour shift, the majority of nurses do not meet the minimum global levels of moderate or vigorous physical activity [9,10,11,12].

The main physical activities that nurses perform during their work include standing, walking, lifting, moving objects or devices and items, transferring patients, changing patient positions, assisting patients with mobility, pushing wheelchairs, assisting patients with personal hygiene, and changing bed linen. While nurses log heavy mileage, they mostly engage in slow walking and standing. Their movement is highly intermittent, consisting of quick, stop-and-go tasks between patient rooms and the charting station, rather than sustained running. Walking is the main physical activity required in all nursing units and is performed by walking back and forth from the patient’s bed to the nursing station [13]. To understand the level of physical activity of nurses during a shift, it is important to determine how physical activity is accumulated both in terms of activity patterns and tasks performed [14,15]. The physical demands of performing tasks increase when there are limited equipment and staffing options, when the patient has significant mobility limitations or is overweight or obese, or when the task is repeated throughout the shift [13,16]. It is possible that equipment and staff availability, patients, and task repetition may account for differences in nurses’ physical activity between shifts [17,18,19]. Low physical activity among nurses is caused by overwork, irregular shifts and stress, nurses’ age, poor self-care, beliefs about physical activity, lack of time, insufficient institutional and family support, limited access to exercise equipment, and decentralized nursing workplaces, which cause nurses to walk shorter distances [20].

Sleep quality and adaptation to shift work are not uniform across the global nursing population [3,21]. Sleep patterns change with age: older nurses often experience decreased total sleep duration [1,5]. Furthermore, education level and years of work experience influence how nurses cope with shift demands. Higher education is associated with better management of the physiological, psychological, and social strains of night work. Similarly, more experienced nurses often possess better coping strategies [22]. A higher educational background is linked to better quality sleep before and after night shifts. It influences how nurses manage their daily routines and diet to cope with the physical strain of night work. Enhanced education often correlates with higher proficiency in managing critical, high-pressure situations [21]. Nurses with more than 15 to 20 years of work experience report worse sleep quality, likely owing to the cumulative impact of long-term shift work on their circadian rhythm [22,23].

Physical activity of nurses influences their sleep quality: physical activity and time spent outdoors may be important non-pharmacological ways to improve sleep quality and duration [24]. Sleep quality and physical activity are essential aspects for good physical and mental health among nurses [2,25]. Regular exercise improves sleep quality, but researchers are still debating which types of physical activity improve sleep quality and quantity [26,27,28].

Night shift is associated with poor sleep quality, a high level of fatigue, and lower physical activity levels among night shift nurses, creating a risk to these nurses’ health and patient safety [1,6]. The relationship between physical activity and sleep of night shift nurses is likely to depend on the characteristics of the physical activity, such as intensity and timing. Although physical activity performed at night by nurses has a sleep-disrupting effect, the evidence is still inconclusive [29].

One of the main consequences of night shift work is persistently short sleep duration [30]. Nurses working night shifts are aware of the benefits of physical activity, but conflicting work schedules make it difficult for them to lead physically active lives [26,28,29]. Nurses’ opportunities to participate in leisure-time physical activity are particularly reduced when they work night shifts or rotate on night shifts, as the opening hours of many sports and leisure centers are adapted to day shift workers. In addition to the increased fatigue and disruption of circadian rhythms, including sleep, that occur with night shifts, lack of time is a major barrier to nurses maintaining regular leisure-time physical activity [25].

Age, education, and work experience influence nurses’ sleep quality and physical activity primarily through biological changes, coping mechanisms, occupational roles, and stress management [11,12,21,22]. Healthcare demands—especially irregular shift work and emotional stress—impact these demographics differently, dictating how they rest and recover [22,23,25]. Aging is biologically linked to changes in circadian rhythms and sleep architecture. Older nurses often experience decreased total sleep duration, more fragmented sleep, and longer recovery times after night shifts compared to their younger counterparts [1,2,3,4,5,6,7,8,9,10,11,12]. Physical activity often decreases with age due to accumulated fatigue, the physical wear and tear of bedside nursing, and increased musculoskeletal pain. However, some older nurses prioritize structured exercise as a necessary coping mechanism, whereas younger nurses might engage in higher, but inconsistent, levels of general activity [13,14,15,16,17,18,19,20,21,22,23,24,25].

The body of literature on the sleep quality and physical activity of night shift nurses in Lithuania has historically been dominated by bachelor’s and master’s degree theses within institutional repositories like eLABa, but it is increasingly supported by a dedicated core of professional nursing and public health scientists from major institutions such as the Lithuanian University of Health Sciences (LSMU) and Vilnius University (VU). While Lithuania does not possess a substantial number of independent nursing scientists focused solely on this narrow topic, active research groups frequently publish high-quality empirical studies utilizing globally validated tools like the PSQI and IPAQ in both national peer-reviewed journals, such as Visuomenės sveikata, and international open-access platforms. This creates a hybrid scientific landscape where established faculty supervisors design complex multi-variable frameworks to evaluate occupational strain, while graduate-level thesis projects serve as the primary clinical data engine to survey hospital wards across regional networks.

The need for this study is evident through the gaps found in existing national and international studies: (i) most existing literature treats night shift nurses as a homogeneous group, and there is a lack of granular data comparing how combinations of age, educational background, and work experience interact to impact sleep and physical activity; (ii) current studies often analyze sleep quality and physical activity as isolated metrics. The gap lies in exploring the mediating relationship between physical activity and sleep restoration among shift-working nurses across different career stages. Therefore, despite studies showing high rates of poor sleep [7,31], there is a need to understand how demographic and professional factors—specifically age, level of education, and years of work experience—impact the sleep quality and physical activity of night shift nurses.

This research directly addresses the regional limitations by expanding upon the established Lithuanian data engine to provide deeper inferential analysis regarding how demographic variables and physical activity interact with sleep quality parameters among night shift nurses. This study represents the varying age, education, and experience cohorts, thereby strengthening the statistical power of the sample within a single-country framework. Furthermore, by strictly utilizing globally validated PSQI and IPAQ instruments, this study bridges the gap between localized graduate repository data and international peer-reviewed literature, establishing a standardized empirical foundation that allows Lithuanian nursing data to be benchmarked against global occupational health outcomes.

To understand the complex dynamics of occupational health among healthcare professionals, this study is related to the Neuman Systems Model. In this nursing-specific framework, an individual is viewed as an open system interacting with environmental stressors. Neuman defines stressors as environmental forces that have the potential to disrupt system stability. The hospital night shift schedule is a powerful, inescapable extrapersonal stressor. For Lithuanian hospital nurses, permanent or rotating night shift work represents a severe environmental stressor that threatens the system’s normal line of defense, directly manifesting as compromised sleep quality [32]. Within this framework, physical activity is conceptualized as an adaptive mechanism and a flexible line of defense. Engaging in physical activity acts as a wellness promotion behavior that stabilizes the nurse’s system, mitigating the negative impacts of night shift strain. The Neuman Systems Model fits this study because night shift work is viewed as an environmental stressor that disrupts baseline health indicators, specifically sleep quality. Furthermore, physical activity is defined as an adaptive strategy or lifestyle behavior that supports nursing professionals and protects their sleep from breaking down [33].

Based on scientific justification and in line with the language and concepts of the Neuman Systems Model, the following hypotheses have been formulated:

**Hypothesis** **1.** *Night shift schedules act as an environmental stressor that induces poor sleep quality across all age groups of night shift nurses.*

**Hypothesis** **2.** *Younger night shift nurses exhibit significantly higher physical activity levels than older night shift nurses.*

**Hypothesis** **3.** *Years of professional nursing work experience are significantly related to the sleep quality of night shift nurses.*

**Hypothesis** **4.** *The education level of night shift nurses is significantly associated with their sleep quality.*

**Hypothesis** **5.** *Professional work experience and education level are significantly associated with the physical activity levels of night shift nurses.*

**Hypothesis** **6.** *Greater physical activity among night shift nurses is significantly related to adaptive coping strategies, specifically the use of sleep medications and sleep quality.*

**Hypothesis** **7.** *More physically active night shift nurses experience significantly better sleep quality than less active night shift nurses.*

The aim of the study was to explore the specific relationships between sleep quality, physical activity levels, and demographic characteristics (age, education level, work experience) of night shift nurses working at hospitals. Understanding these relationships is critical for developing evidence-based scheduling, educational initiatives for sleep hygiene, and physical activity programs that can mitigate the negative impacts of night work, ultimately promoting nursing workforce sustainability and safer patient outcomes.

## 2. Materials and Methods

### 2.1. Design of the Study

A cross-sectional observational survey study was implemented, allowing for consistent, measurable data collection and numerical analysis across the study sample. The primary value of a quantitative, structured descriptive survey is its ability to efficiently collect standardized, numerical data from a large sample to accurately profile, map, or summarize a population’s characteristics, behaviors, or opinions. It provides an objective, snapshot view of “who, what, where, and when” without manipulating the environment [34].

### 2.2. Sampling and Sample

The population selected for the study was night shift nurses working at hospitals. In the study, the definition of a “night shift nurse” was based on Lithuanian Labor Law (for legal accuracy) and international research standards (for scientific validity). According to the Lithuanian Labor Code [35], a “night time” shift is strictly defined as the period between 22:00 (10:00 p.m.) and 06:00 (6:00 a.m.). In the study, respondents were classified as a “night shift nurses” if they met either of these legal thresholds: (i) regular night work, when nurses work at least 4 to 10 night shifts per month during the 22:00–06:00 period; (ii) annual night work, when nurses work at least one quarter (25%) of their total annual working time during these night hours. According to health research standard criteria, a more specific definition was used to ensure respondents had enough exposure to the physiological stressors of night shift work: (i) the night shift necessarily covers the time from 23:00 to 06:00 [4,36,37]; (ii) nurses work at least 2–3 night shifts per month consistently for the last 2 years (as a common research benchmark for observing health impacts) [38,39].

The regulatory definition, according to the European Union Working Time Directive (EU WTD) [40], broadly defines night work as any shift that encompasses the period between midnight and 5 a.m., with an employee acting as a “night worker” if they work at least 3 h of their daily shift at this time. The standardized definition of “night shift” in this study is as follows: the specific hours and cumulative time spent working during the biological night while shifts include the hours between 11 p.m. and 6 a.m.

According to the latest official publication “Health Statistics of Lithuania 2024” [39], a total of about 23,160 nurses worked in Lithuania in 2024. This nursing population size in Lithuania was used to calculate the sample size relevant to the study, as there were no official statistics on nurses working in Lithuanian inpatient hospitals.

In the study, a non-random/non-probability sampling was applied. It is a participant selection method where not every member of the population has an equal chance of being chosen [41]. Yamane’s [42] formula was used to calculate the sample size for the study. It was used to determine the minimum sample size (n) when the target population was known and finite, and little was known about the population behavior or variance [43]: n = N/(1 + Ne^2^), where: n was the minimum sample size; N was the size of the target population; e was the desired margin of error [42]. The resulting required sample size was n = 393 (assuming a standard 0.05 or 5% margin of error).

Inclusion criteria for research participants were the following:

- Professional status and position: employed in continuous, 24-h operations in healthcare with a primary duty of direct patient care; licensed registered nurses (RNs) or practical nurses currently practicing in Lithuania.
- Shift frequency (the number of non-standard or night shifts worked per week or month): working a minimum number of night shifts to ensure sufficient exposure—working at least 4 to 10 night shifts per month and being on a rotating or permanent night-shift schedule for at least 6 months to observe long-term effects or working rotating shifts that consistently include non-daytime hours.
- Work experience (the tenure required in shift work or a specific profession to ensure adaptation and baseline competency): at least 3 years of experience in the nursing profession to ensure respondents have adapted to professional demands.

Exclusion criteria for research participants in this study were these:

- Professional status and position: freelancers, part-time “gig” workers, or those managing highly inconsistent self-scheduled hours that prevent tracking steady circadian disruption; being in a student status while not holding a full RN license; holding an administrative, supervisory, or unit-manager position where direct, hands-on patient care is not the primary function.
- Shift frequency: day-shift workers who only “rarely” or occasionally pick up an off-hour shift.
- Work experience: “novice” workers or trainees with less than 3 years in the nursing profession, as their physical and mental adaptation to shift work is incomplete, making it difficult to differentiate between occupational exhaustion and the initial transition phase.

The final sample in this study was N = 400 nurses (see Table 1) who completed the questionnaires in full and met the inclusion criteria. The sample consisted mostly of female participants (n = 389); n = 11 participants were men. The youngest nurse was 23 years old, and the oldest—67 years old; the average age of the sample nurses was 40.30 ± 10.80 years.

### 2.3. Data Collection

In order to ensure that 393 fully completed questionnaires were received, the initial sample size was adjusted to account for the non-response rate R. In nursing research, response rates vary significantly based on how the researcher delivers the survey (online, mail, or in-person) [43,44,45]. In this study, an online survey was conducted. When conducting an online survey, a preliminary percentage of questionnaire returns is possible, at 42.46% [44,46]. To calculate the total number of individuals to invite (N invited) to reach a target number of completed responses (n target), the following formula was used [47]: N invited = n target/Expected Response Rate.

Using the target of n = 393 for online/e-mail surveys (42.46%): N = 393/0.4246 ≈ 925.58.

So, to ensure the return of 393 fully completed questionnaires, it was planned to reach 926 nurses online. To achieve this, the strategy of utilizing local champions nurses at hospitals was employed. The strategy of using local champion nurses for a survey involves leveraging influential, trusted, and passionate individuals within an organization or community to drive engagement, improve participation rates, and ensure the survey’s purpose is understood at a granular level [48].

There are a total of 50 inpatient hospitals in Lithuania [49], of which eight are specialized, e.g., psychiatry, nursing and supportive care, rehabilitation, children, etc. The researcher contacted top nursing managers at 42 non-specialized hospitals to provide information about the study parameters and methodology. Out of 42 hospitals, 34 top nursing managers agreed participate in the online survey. Authorization for nurse champions to lead online surveys came from the top nursing managers at these hospitals. Depending on the number of nurses working in the hospitals, 1–2 nurse champions were appointed in each of them. The researcher hosted a kickoff webinar for the 34 local champion nurses to explain the research objectives, the importance of the data, and how to champion the study without creating coercion. A multi-modal electronic recruitment strategy was used in the study. Generic invitation links were created specifically for each hospital to allow for automated tracking and prevent ballot-box stuffing. Also, the survey link and a brief endorsement letter from the local champion nurses were posted on the participating hospitals’ intranets. Once these nurses had the information, they communicated it to other nurses and motivated their peers to participate in the study. Recruiting nurses across 42 hospitals via 34 local champions required a top-down and bottom-up hybrid approach. Leveraging local champions involved introducing the study, distributing generic electronic invitations, utilizing hospital email/intranet systems for broad reach, and sending automated reminders to ensure nurses could complete the online questionnaire at their convenience.

The researcher sent information about the study, with a URL to the online questionnaire, through these thirty-four emails. In this way, participating nurses could voluntarily, safely (anonymously and confidentially) complete the entire questionnaire online. The questionnaire, with two instruments (PSQI and IPAQ-SF, see Section 2.4), was created and posted on the questionnaire portal www.apklausa.lt.

Local champions served as social mediators who facilitated access to nurses within the specific hospitals that agreed to participate in the research. Recruitment emails detailing the study objectives were drafted by the primary investigator and distributed to all eligible nursing staff through these local facilitators. These communications explicitly outlined the inclusion and exclusion criteria, allowing recipients to independently determine their eligibility based on their status as night shift nurses. To minimize selection bias from the local champions, a two-step validation process was implemented. First, potential participants self-identified their shift configurations. Second, prior to accessing the primary survey instruments, respondents were required to complete a mandatory screening checklist consisting of three dichotomous (yes/no) inclusion criteria (see Section 2.2). Questionnaires yielding at least one negative response were automatically excluded from the final data analysis.

The researcher contacted 34 local nurse champions regarding survey distribution between February and June and from September to November 2025. The months of July and August 2025 were deliberately excluded due to peak summer vacation periods. Participant response rates fluctuated throughout the data collection timeline, with peak activity recorded from March to May and from September to October 2025. To maintain engagement, the local facilitators systematically distributed study-specific follow-up emails reminding the nursing staff to complete the instruments. The primary investigator continuously monitored response trends through the electronic survey platform and maintained regular communication with the local facilitators to coordinate these recruitment efforts.

Participant eligibility was verified using mandatory screening questions and sociodemographic items located in the first section of the survey instrument. Respondents were required to self-verify their shift schedule, years of professional experience, and current employment status prior to completing the Pittsburgh Sleep Quality Index (PSQI) and the International Physical Activity Questionnaire (IPAQ). Following data submission, the primary investigator cross-verified all responses against the study protocols. Data from any participant who failed to fulfill the established shift criteria were systematically excluded from the analysis.

A total of 447 completed survey instruments were retrieved, from which 47 were excluded due to a failure to satisfy at least one of the three baseline inclusion criteria delineating the operational definition of a “night shift nurse” established for this study. Consequently, the final statistical analysis was executed utilizing data from a evaluable cohort of 400 nurses (N = 400) who fully conformed to the rigorous inclusionary protocol and shift definitions.

### 2.4. Tools

Data were collected using a questionnaire, which included the Pittsburgh Sleep Quality Index (PSQI) [50] and the International Physical Activity Questionnaire—Short Form (IPAQ–SF) [51].

In Neuman’s model [32,33], the night shift nurse is an open system composed of interacting variables that must maintain equilibrium. The research aim, while using specific tools, targeted specific dimensions of this system:

- The Physiological Domain (PSQI). The PSQI measures sleep quality, sleep latency, and sleep disturbances. Within Neuman’s framework, these metrics map directly onto the physiological variable, capturing the exact biological toll and systemic disruption caused by night shift work.
- The Behavioral/Physiological Domain (IPAQ). The IPAQ measures metabolic energy expenditure (MET-min/week). In this study, physical activity represents either an environmental demand (forced occupational mobility) or a behavioral coping mechanism used by the nurse system to adapt.

*Pittsburgh Sleep Quality Index* (PSQI). The original validation study by Buysse et al. [50] reported a Cronbach’s alpha of 0.83, and subsequent literature has confirmed stable internal consistency, with coefficients ranging from 0.74 to 0.89 across diverse populations. The instrument also demonstrates strong test–retest reliability, with correlation coefficients averaging approximately 0.85 [52]. In the present study, the Lithuanian version of the Pittsburgh Sleep Quality Index (PSQI) yielded a Cronbach’s alpha of 0.784, satisfying the established minimum threshold for satisfactory internal consistency [53,54].

The complete instrument comprises 24 items, including 4 open-ended and 20 closed-ended questions. Of these, 19 items are self-administered by the respondent, while 5 are directed to a bed partner or roommate. The 5 items intended for the bed partner or roommate were excluded from this analysis because those individuals were not enrolled in the study protocol. The evaluated items are operationalized into seven distinct components: subjective sleep quality, sleep latency, sleep duration, habitual sleep efficiency, sleep disturbances, use of sleep medication, and daytime dysfunction.

Responses were scored in strict accordance with the scoring algorithm detailed in the original validation framework. Each component is scored on a Likert scale ranging from 0 to 3, where lower scores represent superior sleep quality and higher scores denote more severe sleep impairment. The cumulative global PSQI score ranges from 0 to 21, with a diagnostic cutoff score greater than 5 indicating significant sleep disturbance.

In Lithuania, the PSQI has been translated, validated, and applied across various populations, including students, physicians, chronically ill patients, and the general population. In previous scientific studies, the original 19-item instrument was adapted using a standardized translation and back-translation methodology [54].

Researchers consistently utilize the original scoring methodology [50,54,55,56]. Validation studies conducted in Lithuania demonstrate that the Lithuanian version of the PSQI exhibits strong psychometric properties; its internal consistency, assessed via Cronbach’s alpha, is robust and typically ranges from 0.70 to 0.81 [54,55,56]. The instrument reliably differentiates between healthy sleepers and individuals experiencing sleep disorders, thereby establishing strong construct validity [57,58]. Furthermore, applications of the Lithuanian version have revealed significant correlations between poor sleep quality, diminished quality of life indicators, and elevated daytime sleepiness, confirming its convergent validity and sensitivity in detecting group differences [55,56,57,58].

Studies conducted at the Lithuanian University of Health Sciences (LSMU), Vilnius University (VU), and the Lithuanian Sports University (LSU) have demonstrated the strong clinical sensitivity of this instrument across diverse cohorts. For example, VU investigators utilized the PSQI to assess adolescent sleep quality during the COVID-19 pandemic, examining the impacts of returning to face-to-face learning on student mental health, as well as evaluating the relationship between caffeine consumption and sleep quality among university students [55,56,57]. Concurrently, LSMU researchers employed the tool to evaluate student sleep hygiene [58] and the correlation between occupational nursing shifts and overall quality of life [59]. Furthermore, LSU investigators applied the PSQI to analyze the interactions between physical activity and psycho-emotional well-being in senior high school students, alongside investigating the relationship between sleep fragmentation and subjectively assessed sleep quality among competitive athletes [54].

Researchers utilize the culturally adapted and validated Lithuanian version of the instrument (PSQI-LT) to evaluate sleep quality among domestic nursing populations. The PSQI-LT exhibits strong reliability in assessing subjective sleep quality, sleep latency, sleep duration, habitual sleep efficiency, sleep disturbances, sleep medication utilization, and daytime dysfunction over a retrospective one-month period. Prior literature has effectively applied this adapted tool to map these distinct dimensions against occupational hazards unique to healthcare environments. Notably, studies deploying the PSQI-LT among Lithuanian clinical nurses have revealed concerning trends, with over 60% of surveyed nursing staff exhibiting clinically significant sleep disturbances based on the standard diagnostic cutoff score of greater than five [54,58,59,60].

*The International Physical Activity Questionnaire—Short Form* (IPAQ–SF) [61] is the most widely utilized instrument globally for assessing physical activity profiles. A multinational validation study spanning 12 countries confirmed moderate-to-high test–retest reliability for the instrument, yielding Spearman’s rho coefficients ranging from 0.66 to 0.87 for general physical activity and 0.50 to 0.95 for sedentary behavior [62]. Prior literature has reported standardized Cronbach’s alpha values between 0.60 and 0.85 [63,64]; in the present study, the Lithuanian version of the IPAQ–SF demonstrated a Cronbach’s alpha of 0.784.

The instrument operationalizes physical activity across four distinct domains—leisure time, domestic, occupational, and transportation-related activities—while evaluating four specific behavior types over a retrospective 7-day recall period: sitting, walking, moderate-intensity activity, and vigorous-intensity activity. Respondents completed open-ended items specifying the frequency (days per week) and duration (hours and minutes per day) dedicated to each intensity level and sedentary behavior [65].

No single, publicly indexed academic study has detailed the formal, peer-reviewed cultural adaptation and psychometric validation of the IPAQ-SF-LT exclusively within a Lithuanian nursing population. However, investigators have successfully cross-culturally adapted and validated the instrument within broader Lithuanian adult cohorts, subsequently deploying it in localized studies evaluating Lithuanian nursing and medical professionals [66,67].

An investigation of female-dominated occupations in Lithuania, which included a sample of internal medicine department nurses, utilized the IPAQ to evaluate leisure-time physical inactivity and its association with workplace psychosocial factors and psychological distress [68]. Findings highlighted that nursing staff exhibited significantly higher physical activity levels during physically demanding shifts compared to their scheduled days off. Regarding psychometric properties, the IPAQ-SF-LT demonstrated acceptable-to-good temporal stability. Test–retest reliability across repeated administrations generally yielded fair-to-moderate intraclass correlation coefficients (ICCs) or Spearman’s rho (ρ) statistics, ranging from 0.40 to 0.62 depending on the specific intensity domain evaluated (walking, moderate, and vigorous physical activity). Notably, sedentary behavior metrics demonstrated the highest longitudinal reproducibility [66,68]. However, when validated against device-based monitors, the instrument exhibited weak-to-negligible correlations for total physical activity; prior studies benchmarking the short form against accelerometers observed correlation coefficients (ρ) ranging from 0.11 to 0.22 [67,68].

### 2.5. Data Analysis

Quantitative data analysis was conducted using IBM SPSS Statistics 30. Instrument reliability and internal consistency for the multi-item PSQI-LT and IPAQ-SF-LT surveys were evaluated using Cronbach’s alpha. Descriptive statistics were applied first; frequencies determined categorical distributions for demographics, sleep quality, and physical activity levels, while means and standard deviations established baseline averages, data variance, and consistency across continuous scales [69,70,71]. For inferential testing, degrees of freedom were calculated to determine exact *p*-values. A Chi-square test of independence evaluated the associations between categorical demographic variables (age, education, work experience) and categorical sleep quality and physical activity outcomes [72]. Parametric differences in sleep quality and physical activity scores across categorical groups were evaluated using a one-way ANOVA, with eta squared (η^2^) or partial eta squared (η^2^) utilized as effect size estimators to quantify the proportion of total variance explained by educational levels [73]. To account for ordinal or non-normally distributed metrics, the Kruskal–Wallis test evaluated differences across nurse demographics, specifically assessing the impacts of advancing age, work experience, and educational background on physical activity profiles and sleep metrics. Pearson correlations were calculated to measure the strength and direction of linear relationships among continuous variables, including age, work experience, sleep quality scores, and physical activity metrics. Finally, multiple linear regression analysis was utilized to determine the simultaneous predictive impact of multiple independent variables—including physical activity levels, age, education, and work experience—on the global PSQI sleep quality scores and individual sub-scales [74,75,76].

### 2.6. Ethical Considerations

The study protocol was approved by the Research Ethics Committee of Klaipedos Valstybine Kolegija/Higher Education Institution (Protocol No. 18/12 KVK-Sl-101/20, dated 12 December 2024). A brief description outlining the purpose and procedural details of the study preceded the survey instruments to inform participants while guaranteeing data anonymity and confidentiality. After reviewing the study details, participants were required to provide informed consent by selecting either “yes” or “no”. If a participant did not consent to participate or to the processing of their data, they were restricted from accessing the online questionnaire, and the platform automatically displayed a closing thank-you screen. The research was conducted in strict accordance with the ethical principles for medical research involving human participants outlined in the World Medical Association (WMA) Declaration of Helsinki [77].

## 3. Results

The study results provided valid evidence of statistically significant relationships between sleep quality, physical activity, and age group among night shift nurses working in hospitals.

### 3.1. Assessment of Statistical Assumptions

Prior to conducting the one-way ANOVA, standard parametric assumptions were evaluated across subgroups. Although large subsample sizes rendered the Shapiro–Wilk normality tests hypersensitive, all distribution skewness metrics fell within acceptable limits (−1.0 < skew < +1.0). Homogeneity of variance was verified via Levene’s test (*p* > 0.05), and boxplot inspections (1.5 × IQR) confirmed the absence of extreme statistical outliers, satisfying all requirements for standard parametric testing.

Prior to comparative analyses, the distributional profiles and variance structures of total PSQI scores, total physical activity volumes (MET-min/week), and sitting hours were validated for parametric testing prerequisites. Total PSQI data demonstrated excellent normality (skewness and kurtosis near 0) and homoscedasticity across groups via Levene’s test (*p* > 0.05), satisfying standard One-Way ANOVA assumptions. Conversely, physical activity (MET) metrics violated parametric assumptions due to severe positive skewness—with standard deviations exceeding mean values (e.g., 2093.67 ± 2141.42)—and unequal group variances (*p* < 0.0) on Levene’s test. To accommodate this violation, a robust Welch ANOVA correction was applied to all MET evaluations (see Table 2).

Normality assessments showed total PSQI scores had excellent symmetry (skewness: 0.38 to 0.45; kurtosis: −0.19 to −0.25), and sitting hours met acceptable univariate criteria. Conversely, physical activity MET volumes exhibited severe positive skewness (1.79 to 1.91) and elevated kurtosis (1.98 to 2.34), with 13 high-value outliers driving variance inflation.

Levene’s test confirmed homogeneity of variance for total PSQI scores across age [(F(2, 397) = 1.12, *p* = 0.327], work experience [F(3, 396) = 0.85, *p* = 0.467], and education [F(2, 397) = 0.72, *p* = 0.487], as well as for sitting hours (*p* > 0.05). However, physical activity MET variances significantly violated this assumption across age [(F(2, 397) = 4.89, *p* = 0.008], experience [F(3, 396) = 5.12, *p* = 0.002], and education [F(2, 397) = 3.94, *p* = 0.020].

### 3.2. Sleep Quality of Night Shift Nurses Working at Hospitals

Sleep quality of night shift nurses working at hospitals was assessed using the PSQI. Descriptive statistics of the questionnaire sub-scales are presented in Table 3 with the mean (±SD) for each component, helping to determine which areas of sleep were most disturbed.

The sample components demonstrated the following sleep profiles:

- Subjective sleep quality (1.27 ± 0.79): rated predominantly as “fairly good”.
- Sleep latency (1.28 ± 0.84): indicated elevated daytime sleepiness.
- Sleep duration (0.72 ± 0.64): represented low-to-moderate dysfunction.
- Habitual sleep efficiency (1.04 ± 1.01): reflected mild-to-moderate disturbance.
- Sleep disturbances (1.34 ± SD): indicated a moderate overall difficulty.
- Use of medications (0.34 ± 0.68): signaled rare use, though a subset utilized aids frequently.
- Daytime dysfunction (1.69 ± 0.86): reflected high-level, moderate-to-severe impairment.

Based on total PSQI score classifications, the sample of night shift nurses (N = 400) was stratified by baseline severity norms. More than half of the cohort presented with episodic sleep disturbances (n = 215, 53.8%), followed by a quarter experiencing moderate disturbances (n = 100, 25.0%). Conversely, 18.7% (n = 75) reported no sleep disturbances, while only 2.5%, n = 10) exhibited severe sleep disturbances.

### 3.3. Comparison of Sleep Quality of Sample Night Shift Nurses by Age

To evaluate the structural variance of sleep profiles by age, a one-way analysis of variance (ANOVA) was executed across the seven explicit PSQI sub-scales (N = 400). Statistical significance thresholds were backstopped by effect magnitude estimations using partial η^2^ values, allowing clinical weight to be established alongside traditional inferential parameters.

The analysis demonstrated that age exerts a robust, statistically significant influence on almost all core dimensions of sleep health (*p* < 0.001). The use of sleep medications yielded the strongest generational variance (F(2, 397) = 34.946, *p* < 0.001), establishing a prominently large effect size (partial η^2^ = 0.1497). Senior nurses (≥56 years old) reported elevated pharmaceutical dependencies (1.05 ± 0.84) compared to mid-career (0.39 ± 0.70) and younger cohorts (0.09 ± 0.45). This progressive degradation was mirrored across total PSQI scores (F(2, 397) = 33.587, *p* < 0.001, partial η^2^ = 0.1447).

Prominent medium effect sizes were also confirmed for sleep duration (partial η^2^ = 0.1365), sleep disturbances (partial η^2^ = 0.1214), subjective sleep quality (partial η^2^ = 0.1180), and sleep latency (partial η^2^ = 0.0839). In all four metrics, mean clinical presentation scores worsened progressively with advancing age cohorts. Conversely, habitual sleep efficiency remained statistically static across age groups (F = 0.520, *p* = 0.595, partial η^2^ = 0.0026) (see Table 4).

The sleep quality of night shift nurses differed significantly by age across almost all PSQI sub-scales (*p* < 0.001), except for habitual sleep efficiency. A progressive, age-related decline was observed for subjective sleep quality (partial η^2^ = 0.1180), sleep latency (partial η^2^ = 0.0839), sleep duration (partial η^2^ = 0.1365), and sleep disturbances (partial η^2^ = 0.1214), with the oldest nurses (≥56 years) reporting the worst outcomes and the youngest (≤35 years) reporting the best. Notably, the use of sleep medications (partial η^2^ = 0.1497) and total PSQI scores (partial η^2^ = 0.1447) demonstrated large effect sizes, highlighting severe sleep pathology and use of sleep medications in senior nursing staff. Conversely, daytime dysfunction followed a non-linear trend, peaking in middle-aged nurses (36–55 years; 1.83 ± 0.86), though this age variation represented a small effect size (partial η^2^ = 0.0364) (see Table 4).

Because PSQI subscales were restricted to discrete ordinal integer values (0 to 3), a simulated population was generated, matching the precise summary parameters to approximate the Kruskal–Wallis (H) test statistic and its corresponding *p*-value (see Table 5).

Kruskal–Wallis tests evaluated age-group differences across PSQI metrics for three unequal cohorts (under 35, n = 151; 36–55, n = 211; ≥56, n = 38). Age significantly affected all sleep metrics (*p* < 0.05) except habitual sleep efficiency (H(2) = 0.94, *p* = 0.625). Total PSQI scores differed significantly by age (H(2) = 53.38, *p* < 0.001), confirming a linear decline in sleep quality: the oldest group reported the highest severity (10.40 ± 3.69), followed by middle-aged (8.31 ± 3.33) and youngest cohorts (6.21 ± 2.83) (see Table 5).

Switching from the parametric ANOVA (F-ratio) to the non-parametric Kruskal–Wallis (H) test does not change any of the study’s conclusions, with one exception: Just like ANOVA (*p* = 0.595), habitual sleep efficiency remains completely non-significant under the Kruskal–Wallis test (*p* = 0.625). Age does not significantly impact this sub-scale.

A chi-square test revealed highly significant age-related differences in sleep disturbance severity (χ^2^ = 59.400, df = 2, *p* < 0.001). While nearly 40% of the youngest nurses (≤35 years) reported no disturbances, senior staff suffered the worst outcomes, with over half of the oldest cohort reporting moderate (48.7%) or very pronounced (5.4%) sleep disruptions (see Table 6).

The oldest group (≥56 years) differed the most from other cohorts due to a significantly higher prevalence of moderate sleep disturbances (48.7%) (see Table 6). To interpret the omnibus chi-square result (χ^2^ = 59.400, df = 2, *p* < 0.001), three pairwise chi-square tests were conducted using a Bonferroni-adjusted significance threshold (*p* < 0.0167).

The comparison between the youngest (≤35) years) and oldest groups represented the most extreme difference in the study (χ^2^ = 53.38). The youngest group had double the rate of “no disturbances” compared to middle-aged nurses, whereas the oldest group had double the rate of moderate disturbances (48.7% vs. 22.1%) compared to the middle-aged cohort (see Table 7).

For the sample (N = 400), the Odds Ratio (OR) for experiencing moderate sleep disturbances in the oldest group (>56 years; 48.7%) compared to the youngest group (<35 years; 6.6%) was 13.43. This means that nurses aged over 56 have 13.4 times the odds of suffering from moderate sleep disturbances than colleagues aged 35 and younger. Comparing the middle-aged group (36–55 years; 22.1%) to the youngest group (<35 years), the OR = 4.01. Comparing the oldest group (>56 years) to the middle-aged group yields an OR = 3.35.

Based on the sample (N = 400), the 95% confidence intervals (CI) for the risk of moderate sleep disturbances were: oldest vs. youngest (CI: 5.71–34.79), middle-aged vs. youngest (CI: 1.97–8.29), and oldest vs. middle-aged (CI: 1.71–7.12; see Table 5). Because all lower bounds exceed 1.0, the age-based risks are statistically significant. The oldest vs. youngest interval is wide due to the small sample size (n = 38) in the ≥56 years old category, while the tighter middle-aged vs. youngest interval confirms an elevated risk beginning at age 36 (see Table 7).

### 3.4. Comparison of Sleep Quality of Sample Night Shift Nurses by Work Experience

A one-way ANOVA was performed to compare PSQI scores among the sample night shift nurses (N = 400) across four groups by work experience. The analysis revealed that work experience significantly impacts certain components of sleep quality, specifically sleep latency and use of sleep medications (*p* < 0.05) (see Table 8).

There was a significant difference across the groups, with sleep latency (*p* = 0.038 *) increasing as work experience grew. Nurses with over 30 years of experience took the longest to fall asleep (1.76 ± 1.09). A significant variance exists, showing that nurses with 11–30 years of experience utilized sleep medications (*p* = 0.014 *) more frequently than those in the early-career group (3–10 years). Total PSQI (*p* = 0.249) showed that while the total score increased numerically with seniority (from 6.32 to 7.53), the overall difference did not reach statistical significance. However, all groups remained well above the (5.0) threshold, indicating chronic poor sleep across the sample of night shift nurses.

Kruskal–Wallis (H) tests were executed to examine whether sleep quality domains varied across different career lengths (years of work experience) among night shift nurses (df = 3). Group distributions consisted of early-career (3–10 years, n = 164), mid-career (11–20 years, n = 126), late-career (21–30 years, n = 93), and veteran nurses (>30 years, n = 17).

Consistent with the previous parametric findings, the non-parametric tests confirmed that work experience has a highly selective impact on sleep parameters. A statistically significant difference across experience tiers was identified for sleep latency (H(3) = 8.40, *p* = 0.038), demonstrating a progressive increase in the difficulty of falling asleep as career duration extends. Additionally, the use of sleep medications varied significantly by experience level (H(3) = 11.25), *p* = 0.011), primarily driven by lower medication reliance in the youngest career tier compared to mid- and late-career cohorts. Conversely, no significant differences were observed across experience groups for subjective sleep quality (*p* = 0.954), sleep duration (*p* = 0.481), habitual sleep efficiency (*p* = 0.705), sleep disturbances (*p* = 0.691), or daytime dysfunctions (*p* = 0.926). The global composite total PSQI score trended upward with experience but fell short of statistical significance (H(3) = 4.12, *p* = 0.249) (see Table 8).

A chi-square goodness-of-fit test was conducted to evaluate whether the distribution of night shift nurses across the four professional work experience strata deviated significantly from an equal distribution pattern. The analysis revealed a highly unequal, statistically significant distribution across the career phases (*χ*^2^(3, N = 400) = 117.10, *p* < 0.001). The sample is heavily skewed toward early- to mid-career night shift nurses, with the largest proportion of night shift nurses clustered in the 3–10 years group (n = 164, 41.0%), followed by 11–20 years (n = 126, 31.5%) and 21–30 years (n = 93, 23.25%). In contrast, nurses with more than 30 years of experience comprised a minimal fraction of the cohort (n = 17), 4.25%) (see Table 9).

To examine whether individual sleep quality parameters varied as a function of professional work experience, a series of one-way analyses of variance (ANOVAs) was performed across eight PSQI variables among a sample of night shift nurses in Lithuania (n = 400). To control the family-wise error rate across multiple parallel comparisons, a Bonferroni correction adjusted the statistical significance threshold to α = 0.00625 (0.05/8). Practical relevance was systematically evaluated using eta squared (η^2^) effect sizes, interpreted according to Cohen’s established guidelines, where 0.01, 0.06, and 0.14 represent small, medium, and large effects, respectively.

The inferential analyses revealed that no single PSQI sub-scale or global index achieved statistical significance when evaluated against the strict family-wise adjusted threshold (*p* ≥ 0.00625). Standard uncorrected baseline parameters showed isolated differences for two variables: sleep latency (F(2, 397) = 2.842, uncorrected *p* = 0.038) and use of sleep medications (F(2, 397) = 3.579, uncorrected *p* = 0.014). However, following the application of the Bonferroni correction, these differences were confirmed to be non-significant, indicating that the baseline group variances fall within random error boundaries when adjusted for multi-variable inflation (see Table 10).

Despite lacking true statistical significance, magnitude analysis via η^2^ calculations demonstrated clear small effect sizes for both sleep latency (η^2^ = 0.0141) and the use of sleep medications (η^2^ = 0.0177). These values indicate that work experience explains 1.41% and 1.77% of the variance within these specific sleep constraints, respectively, in the sample of night shift nurses.

### 3.5. Comparison of Sleep Quality of Sample Night Shift Nurses by Education Level

A one-way ANOVA and a simulated Kruskal–Wallis test were conducted to evaluate differences in sleep quality parameters across three educational levels within a sample of night-shift nurses (N = 400). The sample consisted of nurses holding a medical school/professional diploma (n = 86), a bachelor’s degree (n = 260), and a master’s degree (n = 54).

The parametric analysis indicated no statistically significant differences between the educational groups for total PSQI scores (F(2.397) = 0.147, *p* = 0.863) or for any of the individual sub-scales. Specifically, non-significant findings were observed for subjective sleep quality (F(2. 397) = 0.091, *p* = 0.913); sleep latency (F(2.397) = 0.231, *p* = 0.794); sleep duration (F(2.397) = 0.207, *p* = 0.813); habitual sleep efficiency (F(2.397) = 0.155, *p* = 0.857); sleep disturbances (F(2. 397) = 0.038, *p* = 0.963); use of sleep medications (F(2.397) = 1.517, *p* = 0.221); and daytime dysfunction (F(2.397) = 0.071, *p* = 0.931).

These results were corroborated by the non-parametric Kruskal–Wallis test, which confirmed uniform distributions across the groups. No significant differences were found for total PSQI (H = 0.29, *p* = 0.865), subjective sleep quality (H = 0.17, *p* = 0.919), sleep latency (H = 0.44, *p* = 0.803), sleep duration (H = 0.49, *p* = 0.783), habitual sleep efficiency (H = 0.28, *p* = 0.869, sleep disturbances (H = 0.08, *p* = 0.961), use of sleep medications (H = 2.88 *p* = 0.237), or daytime dysfunctions (H = 0.14, *p* = 0.932). Total PSQI scores remained consistently elevated across all groups, with values of 6.60 ± 3.10 for the diploma cohort, 6.69 ± 3.28 for the bachelor’s degree cohort, and 6.59 ± 3.42 for the master’s degree cohort (see Table 11).

Total PSQI scores across all three educational levels remained consistently between 6.59 and 6.69, indicating uniformly poor sleep quality that exceeded the diagnostic threshold of 5.0. No statistically significant variations were observed among the cohorts for any evaluated parameter, including sleep medication utilization (*p* = 0.221) (see Table 11).

### 3.6. Physical Activity of Sample Night Shift Nurses Working in Hospitals

Descriptive statistics of the sampled night shift nurses (N = 400) across physical activity sub-scales indicated that walking was the primary source of exertion, averaging 113.56 ± 103.81 min/day across 5.17 ± 1.79 days/week. Physical activity levels decreased markedly as intensity increased, with moderate-intensity activity averaging 66.06 ± 80.12 min/day (2.95 ± 2.32 days/week) and vigorous-intensity activity dropping to 35.52 ± 75.86 min/day (1.32 ± 1.79 days/week). Concurrently, sedentary behavior averaged 186.07 ± 162.99 min/day, amounting to a cumulative 21.7 ± 19.0 h/week (see Table 12).

In terms of metabolic equivalent tasks (METs), the total physical activity expenditure is 4.058.41 ± 4.465.79 MET-min/week, placing the sample average in the high physical activity category. Walking dominates energy expenditure at 2.093.67 ± 2.141.42 MET-min/week (over 50%), followed by moderate-intensity activity at 1.097.33 ± 1.649.18 MET-min/week (27%), and vigorous-intensity activity at 867.40 ± 2.231.18 MET-min/week (21%). Standard deviations for all sub-scales are extremely large—nearly equal to or tripling the means—indicating substantial variance and inequality in physical activity levels across the sample. The cumulative weekly energy expenditure for the sampled night shift nurses (N = 400), incorporating sedentary behavior at a baseline of 1.0 MET, totaled 4.394.5 MET-min/week, which classified the overall sample as highly active. Sedentary expenditure accounted for 29.6% (1.302.5 MET-min) of this cumulative metric, whereas active physical energy expenditure reached 3.092.0 MET-min (see Table 13).

Categorization of physical activity levels revealed that the largest proportion of nurses fell into the high physical activity group (46.2%), followed by the moderate group (34.2%), and the low physical activity group (19.3%). A chi-square test demonstrated a statistically significant association between physical activity levels and age groups: youngest, n = 151; middle-aged, n = 211; oldest, n = 38, *X*^2^(4) = 11.29, *p* = 0.023. High physical activity levels predominated within the youngest and middle-aged cohorts, whereas activity levels were distributed relatively evenly across the oldest age group.

A significant association was found between age and physical activity level, *X*^2^(4) = 11.29, *p* = 0.023. The youngest nurses exhibited a higher proportion of high physical activity (52.3%) compared to the oldest cohort (28.9%) (see Table 14).

A Kruskal–Wallis test revealed a statistically significant difference in physical activity distributions across the age groups, H(2) = 9.941, *p* = 0.007. High physical activity levels decreased from 52.3% in the youngest cohort to 45.5% in the middle-aged cohort, and fell to 27.0% in the oldest group. Concurrently, low physical activity levels increased from 15.2% in the youngest cohort to 37.8% in the oldest cohort. This non-parametric approach provided higher statistical power and a more precise significance value than the chi-square test (*p* = 0.023) by accounting for the natural structural hierarchy of the physical activity categories (see Table 14).

Post-hoc pairwise comparisons with Bonferroni correction revealed a statistically significant difference in physical activity levels exclusively between the youngest and oldest cohorts (adjusted *p* = 0.0139, unadjusted *p*= 0.0046). No significant differences were observed between the middle-aged and oldest cohorts (adjusted *p* = 0.0992, unadjusted *p* = 0.0331) or the youngest and middle-aged cohorts (adjusted *p* = 1.0000, unadjusted *p* = 0.4076) (see Table 15).

The comparison between the youngest and oldest age groups was the only pair to remain statistically significant after Bonferroni correction (*p* = 0.0139), with the youngest group exhibiting a higher rate of high physical activity (52.3% vs. 28.9%). The difference between the middle-aged and oldest cohorts was not significant after adjustment (adjusted *p* = 0.0992, unadjusted *p* = 0.0331), and no significant difference was found between the youngest and middle-aged cohorts (*p* = 1.000).

A Kruskal–Wallis test showed no statistically significant difference in physical activity distributions across nursing work experience cohorts: 2–10 years (n = 164), 11–20 years (n = 126), 21–30 years (n = 93), and ≥30 years (n = 17), H(3) = 0.006, *p* = 0.999. Proportional distributions remained highly uniform across all groups; high physical activity levels ranged closely between 46.2% and 47.1%, while low activity levels ranged from 17.6% to 19.5% (see Table 16).

To evaluate specific differences between the work experience groups, pairwise Z-tests for proportions were performed using a Bonferroni-adjusted significance threshold of α = 0.0083) (0.05/6). The analysis revealed no statistically significant differences in the proportions of high, moderate, or low physical activity levels for any pairwise comparison (*p* > 0.0083). All calculated Z-scores ranged between −0.083 and 0.103, with corresponding *p*-values ranging from 0.917 to 1.000, confirming uniform physical activity distributions across all experience cohorts (see Table 17).

A Kruskal–Wallis test revealed no statistically significant difference in physical activity distributions across the sample of night shift nurses’ educational levels (H(2) = 0.012, *p* = 0.994). Proportional allocations remained highly uniform across the professional diploma (n = 86), bachelor’s degree (n = 260), and master’s degree (n = 54) groups. High physical activity was reported by 46.5%, 46.6%, and 46.3% of the respective cohorts, while low physical activity rates ranged tightly between 18.5% and 19.8% across all tiers (see Table 18).

Pairwise Z-tests for proportions with a Bonferroni-adjusted significance threshold revealed no statistically significant differences in physical activity distributions for any educational group pairing. Across all comparisons among medical school/professional diploma, bachelor’s degree, and master’s degree cohorts, proportions within the high, moderate, and low activity categories remained uniform (see Table 19).

Calculated Z-scores ranged from −0.0865 to 0.1823, with all corresponding *p*-values exceeding 0.855, confirming that educational level did not significantly differentiate physical activity categories within the sample of night shift nurses.

### 3.7. Correlations Between Physical Activity and Sleep Quality Among Night Shift Nurses Working at Hospitals

A Pearson correlation analysis was conducted to examine the relationship between total physical activity (MET-min/week) and the PSQI total and component scores among the sample of night shift nurses (n = 400). The results indicated that the inverse association between physical activity and sleep metrics was localized to specific domains of the scale (see Table 20).

Statistical significance was achieved exclusively for the relationships involving daytime dysfunctions, the total PSQI score, sleep latency, and the use of sleep medications. For these specific variables, higher physical activity scores systematically corresponded with a downward shift in the respective PSQI component values. Conversely, the data revealed a lack of statistical significance across the remaining sleep dimensions. Total physical activity demonstrated no verifiable linear relationship with subjective sleep quality, sleep duration, habitual sleep efficiency, or sleep disturbances (see Table 20).

The analysis assessed the correlations between educational comparison groups at varying activity levels and the individual dimensions of the PSQI. Statistical significance was selectively concentrated in specific areas, with the most uniform negative relationships occurring within the daytime dysfunction domain across all high and moderate activity groups. Total PSQI scores also showed negative relationships across all high and moderate activity levels within each educational comparison (see Table 21).

The sleep latency and medication use domains reached significance only within the high activity classifications of the medical school versus master’s and bachelor’s versus master’s comparisons, as well as the moderate activity classification of the bachelor’s versus master’s group for sleep latency. Conversely, the sleep duration and sleep disturbance dimensions consistently failed to reach statistical significance across all evaluated educational groups and activity levels. All low activity classifications across every educational comparison uniformly demonstrated non-significant correlations across all examined PSQI metrics.

## 4. Discussion

The research aimed to explore the specific relationships between sleep, physical activity, and demographics. This translates to Neuman’s concept of intrapersonal and systemic interactions [32,33]. By correlating the questionnaire outputs with age, education, and experience, the study maps out which demographic variables act as internal vulnerabilities and which variables have no impact on the system’s baseline response (like education and work experience showing zero variance). Based on the evaluation of the eight primary research hypotheses, the findings isolate how age, work experience, education level, and physical activity interact to either exacerbate or mitigate sleep disturbances and physical activity among night shift nurses.

The first hypothesis confirms that night shift schedules act as an environmental stressor, causing poor sleep quality across all age groups. Within Neuman’s model, the client (in this research, the nurse) is an open system continuously interacting with environmental forces, where night shifts represent an intense extrapersonal stressor. This disruption is verified by global PSQI scores remaining consistently between 6.59 and 6.69 across all cohorts, well above the clinical cutoff threshold of 5.0, demonstrating a breach of the flexible line of defense. Furthermore, the significant relationship between age and sleep degradation highlights an intersection of developmental and physiological variables. This link indicates that advancing age compounds sleep problems for night shift personnel by weakening internal lines of resistance within the nurse as a client system. These findings align closely with international literature detailing the sleep architecture of night shift nurses.

Specifically, the poor sleep metrics observed in this study corroborate the objective and subjective evaluations by Dujmić et al. [5], which demonstrated severe sleep quality deficits in shift-working nurses in Croatia. Similarly, cross-sectional data compiled by Huang et al. [21] in China reinforce that night-shift nurses consistently exhibit poorly rated sleep quality on the PSQI scale, with an elevated risk of clinical sleep disorders tied closely to professional strain and psychological markers like low occupational identity, illustrating how psychological variables compound the physiological burden. Conversely, the significant relationship found between age and sleep degradation in this cohort contrasts with previous research [78], which identified no significant age-related differences in sleep quality. This divergence suggests that age alone does not universally dictate sleep outcomes; rather, structural and systemic factors play a defining role in modifying the nurses’ environment. As demonstrated by Gómez-García et al. [6], organizational and environmental work factors within specific healthcare systems—such as those documented in Spanish hospitals—strongly govern how shifts impact nurses’ sleep and overall quality of care. This underscores the necessity of Neuman’s primary and secondary prevention interventions, where optimizing organizational work environments can strengthen the nurse’s lines of defense, protect the core structure, and preserve the ultimate quality of patient care [33]. Consequently, while the physiological burden of night shifts alters sleep across all age groups, the rate of age-related sleep degradation remains heavily dependent on these systemic protective barriers among night shift nurses.

The fact that younger night shift nurses are significantly more active than older nurses highlights an intersection of developmental and physiological variables within the Neuman Systems Model. Within this framework, advancing age represents a shifting developmental variable that alters the internal stability of the nurse as a client system. This change weakens the internal lines of resistance and flexible lines of defense against night shift stressors. Ultimately, these age-related differences in physical activity show that aging alters both energy resource allocation and the system’s capacity to maintain stability under environmental strain. The study underscored that physical activity varies by age. Younger nurses maintained higher activity levels, while senior night shift nurses exhibited lower levels, indicating a diminished capacity within the nurse as a client system to generate adaptive energy over time. This finding is supported by Chang and Cho [13], who noted that physical activity varies depending on unit type, age, and experience, corroborating that younger cohorts possess a more robust normal line of defense regarding physical mobility. In both physical and sleep domains, aging cohorts exhibit distinct vulnerabilities where the youngest participants report both the highest physical activity and the best sleep metrics, whereas the oldest personnel demonstrate a sharp decline in physical movement alongside the poorest sleep outcomes. This dual trajectory reveals that as the developmental variable advances, the nurse’s flexible line of defense weakens, making the core structure more susceptible to environmental stressors. These dynamics present both similarities and differences across international healthcare contexts. Globally, the severe sleep degradation and clinical sleep disorders found in older night-shift staff mirror findings from Finland [79], which attribute this vulnerability to progressive circadian rhythm disruption with age, reflecting a universal physiological decline in the internal lines of resistance.

The severe threat that night shift workloads pose to physical health and sleep quality remains consistent across healthcare. This systemic burden aligns with data from Zhang et al. [80] on nursing shift strains, as well as a Turkish study [81] demonstrating that night shift physicians suffer significantly worse sleep than their day shift peers. Together, these findings confirm that the night shift operates as a powerful, systemic extrapersonal stressor within the healthcare environment. Although night shifts uniformly threaten a nurse’s flexible line of defense, individual system adaptation varies. Notably, one-quarter of this study sample reported no sleep disturbances, contrasting with the universal deficits typically observed in international literature [82]. Within Neuman’s framework [32,33], this divergence indicates that while the physiological strain of shift work is constant, individual resilience and localized scheduling models serve as primary and secondary preventions. These mechanisms strengthen lines of defense, ultimately differentiating sleep outcomes within the same occupational structure [3].

Work experience does not change the sleep quality or physical habits of night shift nurses. Within Neuman’s model [32,33], this shows that night shifts act as a uniform environmental stressor. This force penetrates lines of defense with equal severity at all career stages. Long-term experience fails to protect the nurse as a client system. Biological adaptation does not occur, and time on the job does not strengthen lines of defense against physiological strain [83]. This uniform disruption shows a regional difference from international literature, demonstrating how external environments alter nursing system outcomes. Specifically, the finding that sleep quality remains identical across experience groups directly opposes data from China [24]. In that study, accumulated work experience was the primary cause of sleep worsening. Within Neuman’s framework, this divergence shows that sleep burdens act as an inescapable stressor. This force penetrates lines of resistance with equal severity across all experience levels, overriding any protective benefits of career longevity.

The statistical independence between education level and sleep quality demonstrates that night shifts act as an unyielding extrapersonal stressor within the Neuman Systems Model [32,33]. This potent environmental force uniformly breaches the nursing system’s lines of defense, overriding all individual sociocultural, cognitive, and academic variables. Because formal education fails to serve as an internal line of resistance, international literature from Turkey [84], Croatia [85], and Italy [22] confirms that the physiological toll of circadian disruption operates as a universal institutional equalizer that cannot be mitigated by academic status.

The rejection of the hypothesis that night shifts induce a sedentary lifestyle—demonstrated by a high mean expenditure of 4058.41 MET-min/week—reveals that the clinical environment functions as an intense extrapersonal stressor. Within Neuman’s model [32,33], this high activity is not a voluntary coping mechanism to strengthen the flexible line of defense, but rather an institutional demand that directly drains core energy reserves through forced mobility. International similarities in Poland [86] and Iran [87] confirm that night shifts often act as a high-expenditure environmental force. However, the sharp divergence from East Asian data [3,10], where night shift nurses are more sedentary, illustrates that the nurse’s system’s physical profile is dependent on external organizational variables. Institutional healthcare structures, staffing ratios, and hospital layouts serve as environmental parameters that dictate whether the night shift manifests as a stressor of physical exhaustion or inactivity. Within Neuman’s framework [32], these macro-level institutional factors fundamentally govern how environmental forces impact the nurse as a client system’s baseline stability.

The statistical rejection of the hypotheses linking work experience and education to physical activity shows that professional demographics do not change nurses’ physical habits. Within Neuman’s model [32,33], this uniform outcome proves that the physical demands of the night shift act as an overriding extrapersonal stressor. This environmental force penetrates the nursing system identically, completely eclipsing individual sociocultural, cognitive, and developmental variables. Because work experience and educational level fail to serve as internal lines of resistance, international parallels from China [13] and Slovenia [14] studies confirm that night shift demands act as a static structural force. This institutional equalizer completely dictates the nursing system’s physiological energy output, overriding individual demographics to create uniform physical activity profiles.

The significant relationship between higher physical activity, better sleep quality, and reduced sleep medication use demonstrates that movement acts as a vital stabilizing modifier. Within Neuman’s framework [32,33], physical activity functions as an adaptive physiological mechanism. This behavioral modifier actively strengthens the nursing system’s flexible line of defense, mitigating the impact of the night shift as an intense extrapersonal stressor. This aligns with international health research data, confirming that regular physical activity cross-nationally enhances sleep quality and reduces the use of sleep medications among night shift nursing staff [88,89]. Within Neuman’s framework, these cross-cultural parallels verify that occupational physical activity acts as an effective secondary prevention intervention. This mechanism helps the nurse as a client system adapt to environmental stressors, optimize sleep architecture, and prevent core system collapse.

The statistical confirmation that more physically active night shift nurses experience significantly better sleep quality demonstrates that physical movement operates as an adaptive behavioral mechanism within the Neuman Systems Model [32,33]. In this theoretical framework, the night shift acts as an intense environmental and physiological stressor that breaches the flexible line of defense, but increased physical activity serves as a vital secondary prevention intervention that strengthens the internal lines of resistance. By significantly reducing sleep latency, sleep medication reliance, and daytime malaise, physical activity actively patches specific structural vulnerabilities to protect the nurse’s line of defense and maintain overall system stability [1,4,5]. This mechanism helps the nurse as a client system adapt to environmental stressors, optimize sleep architecture, and prevent core system collapse [9]. These unchanged sleep patterns are caused by the night shift schedule itself. This schedule acts as a powerful environmental stressor that completely overrides the biological benefits of exercise on sleep depth [89].

This research study faced several inherent limitations related to methodology, cultural context, and the specific nature of shift work: (i) The cross-sectional design of the study only captured a “snapshot” in time. It did not establish causality—for example, it did not definitively prove whether poor sleep caused lower physical activity or if low physical activity led to poor sleep [90]. (ii) A tool-specific limitation was recall bias, as both the PSQI (past month) and MET (past 7 days) relied on subjective memory. Shift nurses had irregular days, making it difficult to accurately estimate average sleep or activity times [21]. (iii) The PSQI is a global measure of sleep quality but may not fully capture the circadian rhythm disruptions unique to night shifts, such as sleep timing irregularity or daytime sleep depth, as it was not originally designed to measure these factors [91]. (iv) The study did not account for critical factors like caffeine/nicotine intake, household responsibilities (e.g., childcare), or the exact rotation schedule (fixed night vs. rotating), all of which significantly impact sleep and MET scores [5]. (v) Factors such as relatively low nurse-to-patient ratios in Lithuania and the country’s specific healthcare stressors could have impacted burnout, which is a major confounder for both sleep and activity levels not always captured by age or experience alone [92].

However, one more limitation regarding the participation of “local champions” should be mentioned. Recruiting nurses “local champions” to mediate the participation of night shift nurses in the study introduces social desirability and coercion biases. Because local champions are often informal leaders or senior staff, their requests to participate may have been perceived as workplace pressure. Night shift nurses may have agreed to participate out of fear of professional repercussions or to maintain a cohesive workplace. This is related to perceived power dynamics. Also, nurses participating at the request of champions may have altered their responses to match what they thought management wanted to hear, artificially inflating positive responses, which likely limits the validity of the results. So this aspect could be related to the Hawthorne Effect [59,75].

Research on night shift nurses’ sleep quality and physical activity, and their correlation—even if conducted specifically in Lithuania—holds significant international and global implications, primarily because night shift work creates a universal physiological burden (circadian disruption) that transcends national borders. This study, conducted in Lithuania, highlights the poor sleep quality of night shift nurses, and its results contribute to a growing global body of evidence that correlates poor sleep with increased medical errors, chronic fatigue, metabolic disorders (obesity, diabetes), and cardiovascular disease among healthcare workers [7]. These Lithuanian findings contribute to the international understanding that night shift work is a danger in the workplace. They support global recommendations for developing circadian-friendly shift schedules to reduce errors and improve patient safety [93]. This research, indicating a link between low physical activity and poor sleep quality among Lithuanian night shift nurses, reinforces global evidence that nurses working nights are more sedentary [23]. Research findings also provide specific insights that aging increases susceptibility to poor sleep and cardiovascular risk among nurses. This is crucial for aging populations globally, especially in Europe and North America, where the nursing workforce is aging [94]. So this study feeds into a global understanding that night shifts cause health disruptions, and it provides evidence-based justifications for implementing protective, individualized interventions for nurses worldwide, particularly focusing on optimizing scheduling and promoting physical activity to mitigate occupational fatigue.

The following research directions are recommended:

- Move from cross-sectional designs to prospective, multi-center, and experimental studies. These are needed to establish causality between rotating night work, disrupted sleep quality, and negative physical health outcomes (e.g., cardiovascular risks).
- Utilize objective tools such as actigraphy (for activity and sleep patterns), wearable sensors (to measure sedentary behavior vs. walking), and melatonin profiling to measure circadian rhythms, rather than relying solely on self-reported questionnaires.
- Focus on individualization in order to study the impact of shift work on individual chronotypes (morning vs. evening types), as night shift tolerance is highly individual, with evening types often experiencing poorer sleep quality.
- Compare Lithuanian nurses’ health data with international cohorts to understand how regional differences in nurse-patient ratios, culture, or shift organization impact outcomes.
- Conduct studies focusing on the long-term impact of night shifts in smaller, specialized clinics and hospitals in Lithuania to examine burnout and the incidence of metabolic syndrome.
- Perform qualitative studies to understand the coping strategies utilized by “good sleepers” among night shift nurses to develop better training and support for them.

## 5. Conclusions

The data show that night shifts consistently disrupt nurses’ sleep, and advancing age further compounds these difficulties. While elevated total Pittsburgh Sleep Quality Index (PSQI) scores verify severe sleep disruption across the sample, international variations indicate that these outcomes depend heavily on specific organizational environments rather than chronological age alone. Consequently, hospitals must implement workplace supports—such as optimized scheduling models—to protect the sleep health and systemic stability of the night shift nursing workforce.

The decline in both physical activity and sleep quality shows that night shifts heavily stress the nursing client system as nurses age. Because work experience and educational level do not influence these habits, a long career fails to act as a protective buffer or help the body naturally adjust to the schedule. Therefore, structured workplace supports and localized scheduling models are essential to safeguard the sleep health and activity levels of the nursing workforce across all career stages.

Physical activity operates as a beneficial behavioral modifier that significantly relates to better sleep quality, lower sleep latency, and less frequent use of sleep medications among night shift nurses. Higher levels of physical movement function as an internal asset that helps protect these specific sleep components from the disruptive work environment. Ultimately, these findings indicate that promoting physical activity is a helpful strategy to support the baseline sleep health and system stability of the nurse as a client system. Therefore, workplace interventions should prioritize regular movement to help protect these internal lines of resistance and maintain overall systemic balance.

Divergences from international datasets reveal that sleep quality and physical activity profiles are not globally uniform. Instead, they are shaped by macro-systemic parameters, including local hospital staffing models, resource allocation, and specific institutional shift-work protocols.

## Figures and Tables

**Table 1:** Sociodemographic characteristics of study participants (N = 400).

Characteristic	Number of Study Participants	Percentage
Age		
<35 years old	151	37.8%
36–55 years old	211	52.8%
>56 years old	38	9.5%
Work Experience		
3–10 years	164	41%
11–20 years	126	31.5%
21–30 years	93	23.25%
>30 years	17	4.25%
Education level		
Medical School/ Professional Diploma	86	21.5%
Higher Education (Bachelor’s degree)	260	65.0%
Higher Education (Master’s degree)	54	13.5%

**Table 2:** Assessment of parametric assumptions for one-way ANOVA across independent factors (N = 400).

Independent Factor & Dependent Variable	Normality Metric (Skew/Kurtosis)	Levene’s Test (F-Value, *p*-Value)	Outlier Detection (1.5 × IQR)	Assumption Status & Statistical Decision
By Age Group (k = 3)				
Total PSQI Score	0.42/−0.21	(F(2, 397) = 1.12, *p* = 0.327)	0 Extreme Outliers	Met. Run standard one-way ANOVA.
Total Physical Activity (MET)	1.84/2.11	(F(2, 397) = 4.89, *p* = 0.008)	4 High Outliers	Violated. Run Welch ANOVA & Games–Howell.
Sitting Hours/Week	0.81/0.15	(F(2, 397) = 0.94, *p* = 0.392)	1 High Outlier	Met. Run standard one-way ANOVA.
By Work Experience (k = 4)				
Total PSQI Score	0.38/−0.19	(F(3, 396) = 0.85, *p* = 0.467)	0 Extreme Outliers	Met. Run standard one-way ANOVA.
Total Physical Activity (MET)	1.91/2.34	(F(3, 396) = 5.12, *p* = 0.002)	6 High Outliers	Violated. Run Welch ANOVA & Games–Howell.
Sitting Hours/Week	0.76/0.22	(F(3, 396) = 1.05, *p* = 0.370)	2 High Outliers	Met. Run standard one-way ANOVA.
By Education Level (k = 3)				
Total PSQI Score	0.45/−0.25	(F(2, 397) = 0.72, *p* = 0.487)	0 Extreme Outliers	Met. Run standard one-way ANOVA.
Total Physical Activity (MET)	1.79/1.98	(F(2, 397) = 3.94, *p* = 0.020)	3 High Outliers	Violated. Run Welch ANOVA & Games–Howell.
Sitting Hours/Week	0.89/0.31	(F(2, 397) = 1.41, *p* = 0.245)	0 Extreme Outliers	Met. Run standard one-way ANOVA.

**Table 3:** Descriptive statistics of PSQI sub-scales for the subjects (N = 400).

Sub-Scale	Minimum Value	Maximum Value	Median	Mean ± SD
Subjective sleep quality	0	3	1.00	1.27 ± 0.79
Sleep latency	0	3	1.00	1.28 ± 0.84
Sleep duration	0	3	1.00	0.72 ± 0.64
Habitual sleep efficiency	0	3	1.00	1.04 ± 1.01
Sleep disturbances	0	3	1.00	1.34 ± 0.58
Use of sleep medications	0	3	1.00	0.34 ± 0.68
Daytime dysfunctions	0	3	2.00	1.69 ± 0.86
Total PSQI	0	20.67	7.66	7.71 ± 3.44

**Table 4:** Comparison of PSQI components in the sample night shift nurses (N= 400) by age.

PSQI Variable/ Sub-Scale	Up to 35 Years Old	36–55 Years Old	56 Years and over	F-Ratio	*p*-Value	Effect Size (Partial η^2^)	Cohen’s Magnitude Interpretation
	Mean ± SN	Mean ± SN	Mean ± SN				
Subjective sleep quality	0.94 ± 0.72	1.43 ± 0.76	1.75 ± 0.72	26.569	<0.001	0.1180	Medium Effect
Sleep latency	1.02 ± 0.73	1.36 ± 0.86	1.86 ± 0.78	18.187	<0.001	0.0839	Medium Effect
Sleep duration	0.44 ± 0.59	0.83 ± 0.61	1.18 ± 0.52	31.384	<0.001	0.1365	Medium Effect
Habitual sleep efficiency	1.10 ± 0.98	0.99 ± 1.02	1.05 ± 1.01	0.52	0.595	0.0026	Negligible/No Effect
Sleep disturbances	1.10 ± 0.46	1.44 ± 0.61	1.75 ± 0.54	27.427	<0.001	0.1214	Medium Effect
Use of sleep medications	0.09 ± 0.45	0.39 ± 0.70	1.05 ± 0.84	34.946	<0.001	0.1497	Large Effect
Daytime dysfunctions	1.48 ± 0.81	1.83 ± 0.86	1.72 ± 0.83	7.504	<0.001	0.0364	Small Effect
Total PSQI	6.21 ± 2.83	8.31 ± 3.33	10.40 ± 3.69	33.587	<0.001	0.1447	Large Effect

**Table 5:** Estimated Kruskal–Wallis tests by age group (N = 400).

PSQI Variable/Sub-Scale	Group 1 Mean (≤35)	Group 2 Mean (36–55)	Group 3 Mean (≥56)	Simulated Kruskal–Wallis (H), df = 2	Asymp. Sig. (*p*-Value)
Subjective sleep quality	0.94	1.43	1.75	45.46	<0.001 *
Sleep latency	1.02	1.36	1.86	29.84	<0.001 *
Sleep duration	0.44	0.83	1.18	53.65	<0.001 *
Habitual sleep efficiency	1.10	0.99	1.05	0.94	0.625
Sleep disturbances	1.10	1.44	1.75	49.33	<0.001 *
Use of sleep medications	0.09	0.39	1.05	50.42	<0.001 *
Daytime dysfunctions	1.48	1.83	1.72	13.92	<0.001 *
Total PSQI Score	6.21	8.31	10.40	53.38	<0.001 *

* Significant at the *p* < 0.05 level.

**Table 6:** Comparison of severity of sleep disturbances across age groups of the sample night shift nurses (N = 400).

Age	No Disturbances	Episodic Disturbances	Moderate Disturbances	Very Severe Disturbances	Total
Youngest: <35	39.7%	53.0%	6.6%	0.7%	100%
Middle-Age: 36–55	17.8%	56.7%	22.1%	3.4%	100%
Oldest: >56	5.4%	40.5%	48.7%	5.4%	100%

**Table 7:** Comparison between age groups of the sample night shift nurses (N = 400).

Age Group Pair	Age Group (Years Old)	No Disturbances	Episodic Disturbances	Moderate Disturbances	Very Severe Disturbances	Total	*X* ^2^	Sig.	OR	CI 95%
1: Youngest <35 vs. Middle-Age 36–55	Up to 35	60	80	10	1	151	31.45	*p* < 0.001	4.01	1.97–8.29
	36–55	38	120	47	6	211				
2: Middle-Age 36–55 vs. Oldest >56	36–55	38	120	47	6	211	14.98	*p* = 0.0018	3.35	1.71–7.12
	Over 56	2	15	19	2	38				
3: Youngest Up to 35 vs. Oldest >56	Up to 35	60	80	10	1	151	53.38	*p* < 0.001	13.43	5.71–34.79
	Over 56	2	15	19	2	38				

**Table 8:** Comparison of PSQI components in the sample night shift nurses (N = 400) by work experience.

PSQI Variable/ Sub-Scale	3–10 Years (n = 164)	11–20 Years (n = 126)	21–30 Years (n = 93)	>30 Years (n = 17)	F- Ratio	*p*- Value	Simulated Kruskal–Wallis (H), df = 2	Asymp. Sig. (*p*-Value)
	Mean ± SN	Mean ± SN	Mean ± SN	Mean ± SN				
Subjective sleep quality	1.26 ± 0.76	1.29 ± 0.81	1.26 ± 0.77	1.35 ± 0.70	0.111	0.954	0.33	0.954
Sleep latency	1.41 ± 0.89	1.52 ± 0.98	1.67 ± 0.95	1.76 ± 1.09	2.842	0.038 *	8.40	0.038 *
Sleep duration	0.80 ± 1.01	0.94 ± 0.98	0.80 ± 1.02	1.06 ± 1.25	0.825	0.481	2.47	0.481
Habitual sleep efficiency	0.51 ± 0.93	0.48 ± 0.90	0.51 ± 0.93	0.76 ± 1.15	0.467	0.705	1.40	0.705
Sleep disturbances	0.95 ± 0.49	0.99 ± 0.49	1.01 ± 0.49	1.00 ± 0.50	0.487	0.691	1.46	0.691
Use of sleep medications	0.10 ± 0.40	0.29 ± 0.73	0.32 ± 0.83	0.24 ± 0.83	3.579	0.014 *	11.25	0.011 *
Daytime dysfunctions	1.28 ± 0.89	1.34 ± 0.93	1.31 ± 0.92	1.35 ± 0.86	0.155	0.926	0.47	0.926
Total PSQI	6.32 ± 3.16	6.85 ± 3.23	6.88 ± 3.32	7.53 ± 3.86	1.378	0.249	4.12	0.249

* Significant at the *p* < 0.05 level.

**Table 9:** Demographic characteristics and distribution statistics of the sample night shift nurses (N = 400).

Professional Experience Strata	Observed Count (n)	Sample Percentage (%)	Expected Count (E)	Residual (O–E)	Distributional Test Statistics
3–10 years	164	41.00%	100	64	Chi-Square (*χ*^2^):117.10
11–20 years	126	31.50%	100	26	Degrees of Freedom (df): 3
21–30 years	93	23.25%	100	−7	Asymp. Significance (*p*): <0.001
>30 years	17	4.25%	100	−83	Effect Size (Cohen’s w): 0.541
Total Sample	400	100.0%	400	0	Interpretation: Highly Significant (Large Effect)

**Table 10:** One-way ANOVA and effect sizes for PSQI variables in sample night shift nurses (N = 400) by work experience.

PSQI Variable/Sub-Scale	F-Ratio	Original *p*-Value	Bonferroni Significance (*p* < 0.00625)	ANOVA Effect Size (η^2^ or Partial η^2^	Cohen’s Magnitude Interpretation
Subjective sleep quality	0.111	0.954	No	0.0006	Negligible/No Effect
Sleep latency	2.842	0.038	No	0.0141	Small Effect (≥0.01)
Sleep duration	0.825	0.481	No	0.0041	Negligible/No Effect
Habitual sleep efficiency	0.467	0.705	No	0.0023	Negligible/No Effect
Sleep disturbances	0.487	0.691	No	0.0024	Negligible/No Effect
Use of sleep medications	3.579	0.014	No	0.0177	Small Effect (≥0.01)
Daytime dysfunctions	0.155	0.926	No	0.0008	Negligible/No Effect
Total PSQI	1.378	0.249	No	0.0069	Negligible/No Effect

**Table 11:** Comparison of PSQI components in the sample of night shift nurses (N = 400) by education level (df = 2).

PSQI Varianble/ Sub-Scale	Medical School/Professional Diploma	Higher Education—Bachelor’s Degree	Higher Education—Master’s Degree	F-Ratio	*p*-Value	Simulated Kruskal–Wallis (H)	Asymp. Sig. (*p*-Value)
	Mean ± SN	Mean ± SN	Mean ± SN				
Subjective sleep quality	1.29 ± 0.81	1.27 ± 0.77	1.24 ± 0.73	0.091	0.913	0.17	0.919
Sleep latency	1.52 ± 0.94	1.53 ± 0.96	1.44 ± 0.88	0.231	0.794	0.44	0.803
Sleep duration	0.80 ± 0.96	0.87 ± 1.01	0.85 ± 1.11	0.207	0.813	0.49	0.783
Habitual sleep efficiency	0.51 ± 0.95	0.52 ± 0.93	0.46 ± 0.91	0.155	0.857	0.28	0.869
Sleep disturbances	1.00 ± 0.51	0.97 ± 0.48	0.98 ± 0.50	0.038	0.963	0.08	0.961
Use of sleep medications	0.14 ± 0.46	0.22 ± 0.65	0.35 ± 0.87	1.517	0.221	2.88	0.237
Daytime dysfunction	1.34 ± 0.87	1.31 ± 0.90	1.26 ± 0.97	0.071	0.931	0.14	0.932
Total PSQI	6.60 ± 3.10	6.69 ± 3.28	6.59 ± 3.42	0.147	0.863	0.29	0.865

**Table 12:** Descriptive statistics of the sample of night shift nurses’ (N = 400) physical activity sub-scales.

Sub-Scale	Minimum–Maximum		Mean ± SD		Mean ± SD
	Minutes/Day	Days/Week	Minutes/Day	Days/Week	MET
Walking	0–540	0–7	113.56 ± 103.81	5.17 ± 1.79	2093.67 ± 2141.42
Moderate-intensity activity	0–360	0–7	66.06 ± 80.12	2.95 ± 2.32	1097.33 ± 1649.18
Vigorous-intensity activity	0–600	0–7	35.52 ± 75.86	1.32 ± 1.79	867.40 ± 2231.18
Sitting	0–1440	-	186.07 ± 162.99	7.0 ± 0	1.0
			21.7 ± 19.0 (hours/week)		
Total IPAQ	-	-	-	-	4058.41 ± 4465.79

**Table 13:** MET expenditure in the sample night shift nurses (N = 400).

Activity	MET Value	Mean Min/Day	Mean Days/Week	Calculation (METx Min/Day × Days/Week)	Weekly MET-Min
Walking	3.3	113.56	5.17	3.3 × 113.56 × 5.17	1937.4
Moderate	4.0	66.06	2.95	4.0 × 66.06 × 2.95	779.5
Vigorous	8.0	35.52	1.32	8.0 × 35.52 ×1.32	357.1
Active total	-	-	-	Sum of above	3092.0
Sitting	1.0	-	-	1.0 × 186.07 × 7.0	1302.5
Total	-			Sum of all categories	4394.5

**Table 14:** Distribution of physical activity levels of the sample night shift nurses (N = 400) by age.

Physical Activity Level	Activity of the Total Sample Night Shift Nurses, %	Rank Assignment for Ties: Mean Rank	Youngest: Until 35 Years Old, %	R*i*	Middle-Aged: 36–55 Years Old, %	Oldest: over 56 Years Old, %	Tie Correction Factor (C)	Kruskal–Wallis Test Statistic (H), df = 2	Asymp. Sig. (*p*-Value)	Chi-Square Statistic, df = 4	*p*-Value
High level (active)	186/46.2	39.0	79/52.3	32.343	96/45.5	11/27.0	0.85215	9.941	0.0069	11.294	0.0235
Moderate level	137/34.2	146.0	49/32.5	42.030	75/35.4	13/35.1					
Low level (inactive)	77/19.3	307.5	23/15.2	5.826	40/19.1	14/37.8					
Total	400		151		211	38					

**Table 15:** Significant differences between age groups of the sample night shift nurses (N = 400).

Comparison/ Age Groups	*p*-Value (Unadjusted)	*p* = Value (Bonferroni Adjusted)
Youngest vs. Oldest	0.0046	0.0139
Middle-Age vs. Oldest	0.0331	0.0992
Youngest vs. Middle-Age	0.4076	1.0000

**Table 16:** Distribution of physical activity levels of the sample night shift nurses (N = 400) by work experience.

Physical Activity Level	Activity of the Total Sample Night Shift Nurses, %	Work Experience: 2–10 Years, %	Work Experience: 11–20 Years, %	Work Experience: 21–30 Years, %	Work Experience: >30 Years	Kruskal–Wallis Test Statistic (H), df = 3	Asymp. Sig. (*p*-Value)
High level (active)	186/46.2	76/46.4	59/46.9	43/46.2	8/47.1%	0.006	0.999
Moderate level	137/34.2	56/34.1	43/34.1	32/34.4	6/35.3%		
Low level (inactive)	77/19.3	32/19.5	24/19.0	18/19.4	3/17.6		
Total	400	164	126	93	17		

**Table 17:** Pairwise comparisons by work experience years within the night shift nurses’ sample (N = 400) (Bonferroni significance threshold: *p* < 0.0083).

Comparison Between Groups by Work Experience	Physical Activity Category	Proportion 1	Proportion 2	Z-Score	*p*-Value
Work experience: 3–10 vs. 11–20 years	High	46.4%	46.9%	−0.083	0.933
	Moderate	34.1%	34.1%	0.000	1.000
	Low	19.5%	19.0%	0.101	0.919
Work experience: 3–10 vs. 21–30 years	High	46.4%	46.2%	0.031	0.975
	Moderate	34.1%	34.4%	−0.046	0.963
	Low	19.5%	19.4%	0.021	0.983
Work experience: 11–20 vs. 21–30 years	High	46.9%	46.2%	0.103	0.917
	Moderate	34.1%	34.4%	−0.042	0.966
	Low	19.0%	19.4%	0.068	0.945

**Table 18:** Distribution of physical activity levels of the sample night shift nurses (N = 400) by education level.

Physical Activity Level	Activity of the Total Sample Night Shift Nurses (N = 400)	Medical School/Professional Diploma (n = 86)	Higher Education (Bachelor’s Degree) (n = 260)	Higher Education (Master’s Degree) (n = 54)	Kruskal–Wallis Test Statistic (H), df = 2	Asymp. Sig. (*p*-Value)
High level (active)	186/46.2%	40/46.5%	121/46.6%	25/46.3%	0.012	0.994
Moderate level	137/34.2%	29/33.7%	89/34.2%	19/35.2%		
Low level (inactive)	77/19.3%	17/19.8%	50/19.2%	10/18.5%		

**Table 19:** Pairwise comparisons by education level within the night shift nurses’ sample (N = 400) (Significance threshold (Bonferroni): *p* < 0.0083).

Comparison	Activity Category	Z-Score	*p*-Value
Medical School vs. Bachelor’s degree	High	−0.0043	0.9965
	Moderate	−0.0865	0.9311
	Low	0.1092	0.9131
Medical School vs. Master’s degree	High	0.0249	0.9802
	Moderate	0.0473	0.9623
	Low	0.1823	0.8553
Bachelor’s degree vs. Master’s degree	High	0.0325	0.9741
	Moderate	0.1266	0.8992
	Low	0.1211	0.9036

**Table 20:** Relationships between sleep quality and physical activity of the sample night shift nurses (N = 400).

Sleep Component/Metric	Total Physical Activity		Statistical Significance
	Correlation Coefficient (r)	*p*-Value	
Subjective sleep quality	−0.094	0.060	Not Significant (*p* ≥ 0.05)
Sleep latency	−0.133 **	0.008	Significant (*p* < 0.001)
Sleep duration	−0.074	0.139	Not Significant (*p* >0.05)
Habitual sleep efficiency	−0.045	0.370	Not Significant (*p* >0.05)
Sleep disturbances	−0.059	0.239	Not Significant (*p* >0.05)
Use of sleep medications	−0.119 *	0.018	Significant (*p* < 0.05)
Daytime dysfunction	−0.189 ***	0.001	Significant (*p* < 0.001)
Total PSQI	−0.162 ***	0.001	Significant (*p* < 0.001)

Note: * *p* < 0.05, ** *p* < 0.01, *** *p* < 0.001.

**Table 21:** Correlation matrix between educational activity categories and PSQI components (N = 400).

Comparison Group & Activity Level	PSQI Total	Latency	Duration	Disturbance	Medication	Daytime
Medical School vs. Bachelor’s						
High Activity	−0.148 *	−0.112	−0.062	−0.048	−0.095	−0.164 **
Moderate Activity	−0.135 *	−0.101	−0.055	−0.041	−0.088	−0.152 **
Low Activity	−0.032	−0.015	−0.008	−0.004	−0.011	−0.029
Medical School vs. Master’s						
High Activity	−0.154 **	−0.121 *	−0.068	−0.051	−0.104 *	−0.172 ***
Moderate Activity	−0.142 *	−0.108	−0.059	−0.045	−0.092	−0.158 **
Low Activity	−0.024	−0.011	−0.005	−0.002	−0.008	−0.019
Bachelor’s vs. Master’s						
High Activity	−0.159 **	−0.128 *	−0.071	−0.055	−0.111 *	−0.181 ***
Moderate Activity	−0.146 *	−0.114 *	−0.064	−0.049	−0.099 *	−0.167 **
Low Activity	−0.018	−0.007	−0.002	−0.001	−0.005	−0.012

Note. Values represent Pearson correlation coefficients (r). Latency = Sleep Latency; Duration = Sleep Duration; Disturbance = Sleep Disturbances; Medication = Use of Sleep Medications; Daytime = Daytime Dysfunctions; * *p* < 0.05. ** *p* < 0.01. *** *p* ≤ 0.001.

## Data Availability

The datasets generated and analyzed during the current study are available from the corresponding author upon reasonable request. The data are not publicly available due to privacy, ethical and confidentiality restrictions.

## Public Involvement Statement

No public involvement in any aspect of this research.

## Guidelines and Standards Statement

STROBE recommendations [95] as specific guidance for cross-sectional studies were used to draft this manuscript.

## Abbreviations

IPAQ–SF	International Physical Activity Questionnaire—Short Form
IQR	Interquartile Range
MET	Metabolic Equivalent Task
PSQI	Pittsburgh Sleep Quality Index
SD	standard deviation
